# Concerning Food

**Published:** 1890-04

**Authors:** 


### CONCERNING FOOD.

We have, in the different varieties of food we eat, the phosphates, the nitrates, and the carbonates. The first makes *brain;* the second makes *bone* and *muscle;* the last makes *fat.*

By analysis of the *leading* articles of *food*, we have the following results:

| | Phos. parts. | Nit. parts. | Carb. parts. | | Phos. parts. | Nit. parts. | Carb. parts. |
|---|---|---|---|---|---|---|---|
| Beef gives........5 | | 15 | 20 | Corn ..............1 | | 12 | 72 |
| Mutton ............3 | | 12 | 40 | Buckwheat ........1 | | 8 | 15 |
| Lamb............ .3 | | 12 | 35 | Barley.............3 | | 17 | 10 |
| Pork .... .........1 | | 10 | 50 | Oats...............3 | | 17 | 66 |
| Veal... ...........4 | | 16 | 16 | Beans .............4 | | 24 | 57 |
| Salmon ............7 | | 20 | 10 | Peas...............3 | | 22 | 60 |
| Oysters............2 | | 10 | 10 | Rice...............½ | | 6 | 80 |
| Codfish .......... .6 | | 14 | 5 | Potatoes ..... ....½ | | 1 | 22 |
| Eggs ..............5 | | 16 | 0 | Turnips...........½ | | 1 | 21 |
| Wheat ............2 | | 15 | 69 | Cabbage .... ......3 | | 20 | 46 |
| Rye ... .......... 2 | | 12 | 72 | Butter.............0 | | 0 | 100 |

